# Multimodal functional imaging and clinical correlates of pain regions in chronic low-back pain patients treated with spinal cord stimulation: a pilot study

**DOI:** 10.3389/fnimg.2024.1474060

**Published:** 2024-09-27

**Authors:** Yazan Shamli Oghli, Arjun Ashok, Steven Glener, Isaiah Ailes, Mashaal Syed, Ki Chang Kang, Sara Naghizadehkashani, Islam Fayed, Feroze B. Mohamed, Kiran Talekar, Laura Krisa, Chengyuan Wu, Caio Matias, Mahdi Alizadeh

**Affiliations:** ^1^Sidney Kimmel Medical College, Thomas Jefferson University, Philadelphia, PA, United States; ^2^Jefferson Integrated Magnetic Resonance Imaging Center, Department of Radiology, Thomas Jefferson University, Philadelphia, PA, United States; ^3^Department of Neurosurgery, Thomas Jefferson University, Philadelphia, PA, United States; ^4^Department of Neurosurgery, Cooper University Health Care, Camden, PA, United States; ^5^Department of Occupational Therapy, Thomas Jefferson University, Philadelphia, PA, United States

**Keywords:** spinal cord stimulation, chronic low-back pain, resting-state functional MRI (rs-fMRI), pseudo-continuous arterial spin labeling (PCASL), pain Neuromatrix

## Abstract

**Objective:**

Spinal cord stimulation (SCS) is an invasive treatment option for patients suffering from chronic low-back pain (cLBP). It is an effective treatment that has been shown to reduce pain and increase the quality of life in patients. However, the activation of pain processing regions of cLBP patients receiving SCS has not been assessed using objective, quantitative functional imaging techniques. The purpose of the present study was to compare quantitative resting-state (rs)-fMRI and arterial spin labeling (ASL) measures between SCS patients and healthy controls and to correlate clinical measures with quantitative multimodal imaging indices in pain regions.

**Methods:**

Multi-delay 3D GRASE pseudo-continuous ASL and rs-fMRI data were acquired from five patients post-SCS with cLBP and five healthy controls. Three ASL measures and four rs-fMRI measures were derived and normalized into MNI space and smoothed. Averaged values for each measure from a pain atlas were extracted and compared between patients and controls. Clinical pain scores assessing intensity, sensitization, and catastrophizing, as well as others assessing global pain effects (sleep quality, disability, anxiety, and depression), were obtained in patients and correlated with pain regions using linear regression analysis.

**Results:**

Arterial transit time derived from ASL and several rs-fMRI measures were significantly different in patients in regions involved with sensation (primary somatosensory cortex and ventral posterolateral thalamus [VPL]), pain input (posterior short gyrus of the insula [PS]), cognition (dorsolateral prefrontal cortex [DLPC] and posterior cingulate cortex [PCC]), and fear/stress response (hippocampus and hypothalamus). Unidimensional pain rating and sensitization scores were linearly associated with PS, VPL, DLPC, PCC, and/or amygdala activity in cLBP patients.

**Conclusion:**

The present results provide evidence that ASL and rs-fMRI can contrast functional activation in pain regions of cLBP patients receiving SCS and healthy subjects, and they can be associated with clinical pain evaluations as quantitative assessment tools.

## Introduction

Chronic low-back pain (cLBP) is a very common debilitating condition, associated with several etiologies such as neuropathic pain, lumbar spinal stenosis, spondylosis, degenerative disc disease, and more (Last and Hulbert, 2009). It is associated with reduced sleep quality, quality of life, anxiety and depression, and workplace disability, with estimates of economic impact of up to $90.6 billion annually in the United States (Last and Hulbert, 2009; Andersson, 1999). The average primary care physician will see one low-back pain patient per week, and the lifetime prevalence in the United States is 65–80% (Last and Hulbert, 2009; Urits et al., 2019).

One potential surgical option for persistent spinal pain syndrome (Christelis et al., 2021) is spinal cord stimulation (SCS), which aims to stimulate either the dorsal columns or dorsal horns to reduce pain through a number of distinct mechanisms (Caylor et al., 2019). Magnetic resonance imaging (MRI) of the lumbar spine is typically used in the clinical work-up of cLBP, but brain MRIs have also been used to study chronic pain (Davis and Moayedi, 2013). In particular, resting-state functional MRI (rs-fMRI) may be used to assess aberrant synchronous activity in functional networks and oscillatory activity in regions of interest, which may reflect how pain is produced and modulated in chronic pain patients. A rs-fMRI study assessing the amplitude of low-frequency fluctuations (ALFF) and an rs-fMRI index of regional neural activity found that cLBP patients displayed increased ALFF in the supplementary motor area (SMA), anterior cingulate cortex (ACC), insula, and various limbic structures compared to healthy controls (HCs) (Zhang et al., 2019). Similarly, perfusion imaging techniques such as pseudo-continuous arterial spin labeling (PCASL) can provide information based on regional cerebral blood flow (CBF), particularly slowly fluctuating (ongoing/chronic) pain that patients may be experiencing (Loggia et al., 2019; Zhou et al., 2022). A study assessing PCASL-derived CBF in chronic pelvic pain syndrome patients found that patient clinical pain scores were correlated with CBF in the ACC and dorsal lateral prefrontal cortex (DLPC) in patients who responded to treatment (Weisstanner et al., 2017). However, there have been no PCASL studies assessing cLBP patients who have received any treatment, particularly SCS.

A rs-fMRI study assessing the effects of SCS on persistent spinal pain syndrome patients found that it leads to increased connectivity within the salience network associated with pain processing, particularly the connectivity of insular structures to the prefrontal cortex (De Groote et al., 2020). Although functional connectivity findings from this study suggest that synchronous activity in the brain networks of pain patients may be altered by SCS, there have been no studies that have investigated regional, rs-fMRI-derived blood–oxygen level-dependent (BOLD) activation of cLBP patients receiving SCS.

The purpose of the present pilot study is to quantify the changes in the pain Neuromatrix of cLBP patients implanted with SCS using high-resolution, multimodal functional brain imaging and correlate these alterations in functional and perfusion imaging to clinical scores of pain. This will provide crucial data on the efficacy of assessing potential central plasticity associated with cLBP patients implanted with SCS and will provide a framework for the use of multimodal imaging for prognostic use and the study of the potential supraspinal impact of SCS.

## Methods

### Participants

Five cLBP patients (one female) with implanted SCS were compared to five healthy control subjects (two females). Patient information (ex. demographics, cLBP onset, pharmacological pain control) is presented in Table 1. Patients were on stable doses of pain medications and SCS parameters for at least 3 months prior to image acquisition. Informed consent was obtained from all subjects in this study. This study was approved by the Thomas Jefferson University Hospital Institutional Review Board, and all methods were performed in accordance with the Declaration of Helsinki.

**Table 1:** Patient demographic data.

**Patient identification**	**Age**	**Duration of cLBP relative to MR imaging (months)**	**SCS implantation-to-imaging time (months)**	**Time of imaging**	**Pharmacological control of pain**	**Morphine equivalent dose/day (if applicable)**
P1 (male)	55	43	12	September 2021	Acetaminophen, amitriptyline, clonazepam, cyclobenzaprine, pregabalin	N/A
P2 (male)	56	31	6	June 2022	Gabapentin, tizanidine	N/A
P3 (male)	56	122	4	February 2023	Acetaminophen, pregabalin	N/A
P4 (female)	52	120	5	January 2023	Acetaminophen, hydrocodone-acetaminophen	7.5 every 4 h as needed.
P5 (male)	60	130	6	February 2023	Oxycodone-acetaminophen	15

Data on all five cLBP patients implanted with SCS. Includes age, cLBP onset, implantation of stimulation devices, time of brain imaging, and pharmacological pain interventions concurrently used. Mean ± SD = 55.8 ± 2.86 years.

### Image acquisition

All subjects were scanned using a 3T Siemens Prisma MRI scanner (Siemens Healthcare, Erlangen, Germany), with a transmit/receive (Tx/Rx) head coil. Appropriate MRI safety measures were undertaken to scan the subjects. Patients were scanned with the SCS device switched off. All subjects were asked to relax and think of nothing in particular during the rs-fMRI scan.

### Imaging parameters

Axial 3D magnetization prepared rapid gradient echo imaging (MPRAGE). A structural scan was acquired at a 0.9 × 0.9 × 1.5 mm^3^ voxel size, number of averages = 1, repetition time (TR) = 1,660 ms, echo time (TE) = 2.47 ms, inversion time (TI) = 836 ms, radiofrequency flip angle = 8°, and field-of-view (FOV) = 25 cm. The total acquisition time was 5.62 min. Resting-state fMRI images were acquired axially (35 slices) at a 3.5 × 3.5 × 3.5 mm^3^ voxel size, number of averages = 1, 300 volumes, TR = 2,000 ms, TE = 26 ms, flip angle = 90°, and FOV = 25 cm. The total acquisition time was 10 min. Multi-delay 3D gradient and spin echo (GRASE) PCASL was acquired using five post-label delays (0.5/1.0/1.5/2.0/2.5 s), at a voxel size of 2.5 × 2.5 × 3.0 mm^3^. The acquisition parameters were as follows: number of averages = 1, TR = 4,100 ms, TE = 36.48 ms, TI = 1,800 ms, bolus duration = 700 ms, and 13 pairs of tags and control for each delay. The total acquisition time was 7.18 min.

### Image processing

Resting-state fMRI images were preprocessed in MATLAB 2023a (MathWorks, Natick, MA, USA) using the Data Processing and Analysis for Brain Imaging (DPABI) package (Yan et al., 2016). Main steps included the conversion of Digital Imaging and Communications in Medicine (DICOM) to Neuroimaging Informatics Technology Initiative (NIFTI) files, image realignment, T1 image co-registration to functional images, slice timing correction, nuisance covariates (white matter, cerebrospinal fluid, and global motion) regression, head movement correction using Friston 24 model, and spatial normalization. Frequencies were bandpass filtered in the range of 0.01–0.1 Hz, and individual data were normalized to a Montreal Neurological Institute template and smoothed using a full width at half maximum degree of 7 × 7 × 7 mm^3^. From preprocessed resting-state data, four quantitative maps were derived, namely ALFF, fractional ALFF (fALFF), degree of centrality (DC), and regional homogeneity (ReHo) maps for each individual.

From PCASL data, the quantitative maps of relative CBF, cerebral blood volume (CBV), and arterial transit time (ATT) were derived for each subject using CereFlow software (Translational MRI, LLC, Los Angeles, CA) as described in previous studies (Hu et al., 2019; Benninger et al., 2022).

### Pain-related region-based analysis

**Figure 1:**
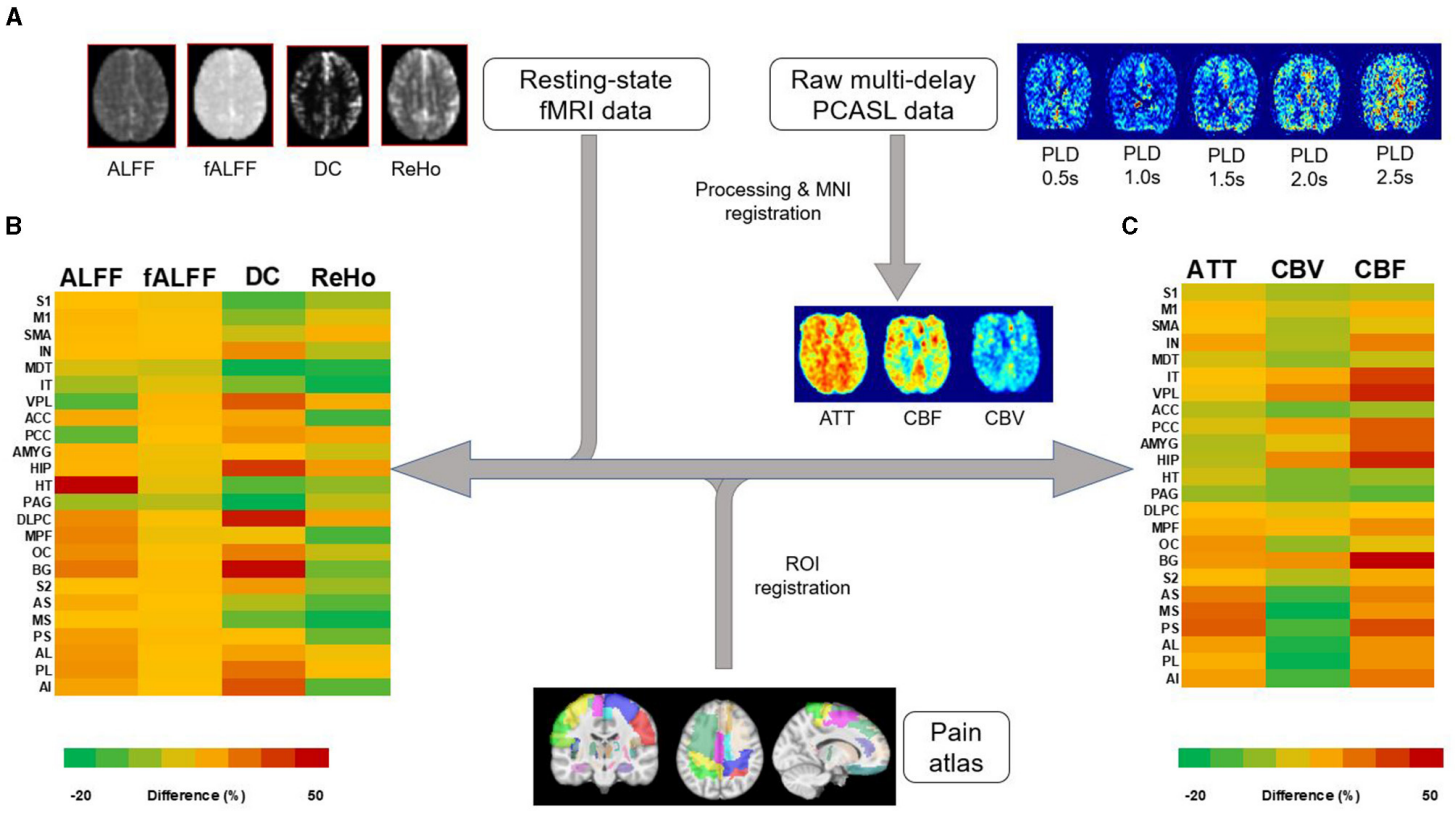
Image processing workflow of rs-fMRI and PCASL data. **(A)** Processing of resting-state fMRI and multi-delay PCASL data was followed by registration of an MRI atlas, representing 23 brain regions associated with the pain Neuromatrix, into individual patient and healthy control MRI space. Percent differences between patient and healthy control measures are represented as a color heatmap for **(B)** rs-fMRI measures: amplitude of low-frequency fluctuations (ALFF), fractional amplitude of low-frequency fluctuations (fALFF), degree of centrality (DC), and regional homogeneity (ReHo). **(C)** A similar heatmap was created for PCASL measures: arterial transit time (ATT), cerebral blood volume (CBV), and cerebral blood flow (CBF). Left and right regions are combined. S1, primary somatosensory cortex; M1, primary motor cortex; SMA, supplementary motor area; IN, insula; MDT, mediodorsal thalamus; IT, intralaminar thalamus; VPL, ventral posterolateral thalamus; ACC, anterior cingulate cortex; PCC, posterior cingulate cortex; AMYG, amygdala; HIP, hippocampus; HT, hypothalamus; PAG, periaqueductal gray; DLPC, dorsolateral prefrontal cortex; MPF, medial prefrontal cortex; OC, orbitofrontal cortex; BG, basal ganglia; S2, secondary somatosensory cortex; AS, anterior short gyrus; MS, middle short gyrus; PS, posterior short gyrus; AL, anterior long gyrus; PL, posterior short gyrus; AI, anterior inferior gyrus.

To examine the specific brain areas involved with the pain Neuromatrix, 26 regions of interest (ROI) from a custom automated anatomical labeling (AAL) atlas (Table 2) were extracted and registered for each patient's rs-fMRI and PCASL data using the Medical Image Registration Toolkit (mirtk.github.io). The regions were comprised of structures detailed in the pain Neuromatrix (Ailes et al., 2023). These were then co-registered into patient space, and average values of rs-fMRI and PCASL maps were collected per ROI for all subjects and were compared between cLBP patients and HCs (Figure 1).

**Table 2:** Pain Neuromatrix atlas regions of interest.

**Region**	**Abbreviation**	**Region**	**Abbreviation**
Primary somatosensory cortex	S1	Periaqueductal gray	PAG
Primary motor cortex	M1	Dorsolateral prefrontal cortex	DLPC
Supplementary motor area	SMA	Medial prefrontal cortex	MPF
Insula	IN	Orbitofrontal cortex	OC
Mediodorsal thalamus	MDT	Basal ganglia	BG
Intralaminar thalamus	IT	Secondary somatosensory cortex	S2
Ventral posterolateral thalamus	VPL	Anterior short gyrus	AS
Anterior cingulate cortex	ACC	Middle short gyrus	MS
Posterior cingulate cortex	PCC	Posterior short gyrus	PS
Amygdala	AMYG	Anterior long gyrus	AL
Hippocampus	HIP	Middle long gyrus	ML
Hypothalamus	HT	Posterior long gyrus	PL

Regions of interest within an AAL atlas were registered into both patient and HC space and used to measure indices of functional imaging using rs-fMRI and PCASL measures.

### Clinical evaluations

Several different qualitative self-assessments were collected for each cLBP patient on the day of image acquisition. These are the Visual Analog Scale (VAS), Numerical Pain Rating Scale (NPRS), Central Sensitization Inventory (CSI), Oswestry Disability Index (ODI), Pain Catastrophizing Scale (PCS), Pittsburgh Sleep Quality Index (PSQI), and the Hospital Anxiety and Depression Scale (HADS).

Pain intensity was assessed using a VAS from 0 to 10 for current pain and NPRS, which averages the patient's worst, current, and lowest pain in the past day. One patient was not administered the pain VAS due to an oversight in providing the assessment. Pain sensitization was assessed using the CSI, with a score of >40 indicating symptoms of central pain sensitization.

We also administered assessments looking at the degree of disability, quality of life, as well as anxiety and depression. The ODI, which determines a patient's self-reported disability due to their pain, ranges from 0 to 5 for each question. A score of 5 indicates complete disability. The PCS assesses the extent to which pain elicits negative thoughts and feelings, with scores ranging from 0 to 52. A score >30, represents significant pain catastrophizing. The PSQI evaluates sleep quality and its impact on daily activities through various questions, with total scores ranging from 0 to 21. A higher score indicates a worse sleep quality. Finally, the HADS provides information on anxiety and depression subscales, with a score of >8 of 21 indicating significant symptoms of anxiety and/or depression.

### Statistics

All statistics were computed using John's Macintosh Project (JMP) Version 17 (SAS Institute Inc., Cary, NC, USA). Imaging variables per ROI were normalized using the Box–Cox transformation method for both rs-fMRI and PCASL quantitative maps before comparisons between patients and HCs (Sakia, 1992). Two-sample *t*-tests were used to compare the means for the variables between patients and HCs, using *p* < 0.05. False discovery rate (FDR) correction was applied to reduce the risk of multiple-comparison false positive errors using *t*-tests. To explore the association between subjective clinical measures and objective quantitative imaging, linear regression analyses were performed, correlating patient-level quantitative maps for each ROI with clinical assessment scores. Significant associations were those with a *p* < 0.01.

## Results

Clinical evaluation data for all cLBP patients implanted with SCS are summarized in Table 3. The VAS was collected for 4/5 patients, and the NPRS, CSI, ODI, PCS, PSQI, and HADS were collected for all five patients. The mean age ± standard deviation for cLBP patients was 55.8 ± 2.86 years. The mean age ± standard deviation for healthy controls (HCs) was 48.40 ± 5.41 years.

**Table 3:** Clinical evaluation results of cLBP patients implanted with SCS.

**Patient identification**	**VAS (/10)**	**NPRS (/10)**	**CSI (/100)**	**ODI (/50)**	**PCS (/52)**	**PSQI (/21)**	**HADS (anxiety/depression) (/21 for each)**
P1	N/A	3.6	26	6	9	14	6/4
P2	4	5	37	21	5	9	3/1
P3	2	2	38	5	19	8	5/4
P4	7	4	36	30	3	8	3/2
P5	9	8	58	28	22	17	12/10

Clinical evaluation of patients was completed on the day of imaging. VAS, Visual Analog Scale; NPRS, Numerical Pain Rating Scale; CSI, Central Sensitization Inventory; ODI, Oswell Disability Index; PCS, Pain Catastrophizing Scale; PSQI, Pittsburgh Sleep Quality Index; HADS, Hospital Anxiety and Depression Scale.

^*^VAS for P1 was not collected.

### Comparison of cLBP patients implanted with SCS to healthy control subjects

#### Resting-state fMRI

**Figure 2:**
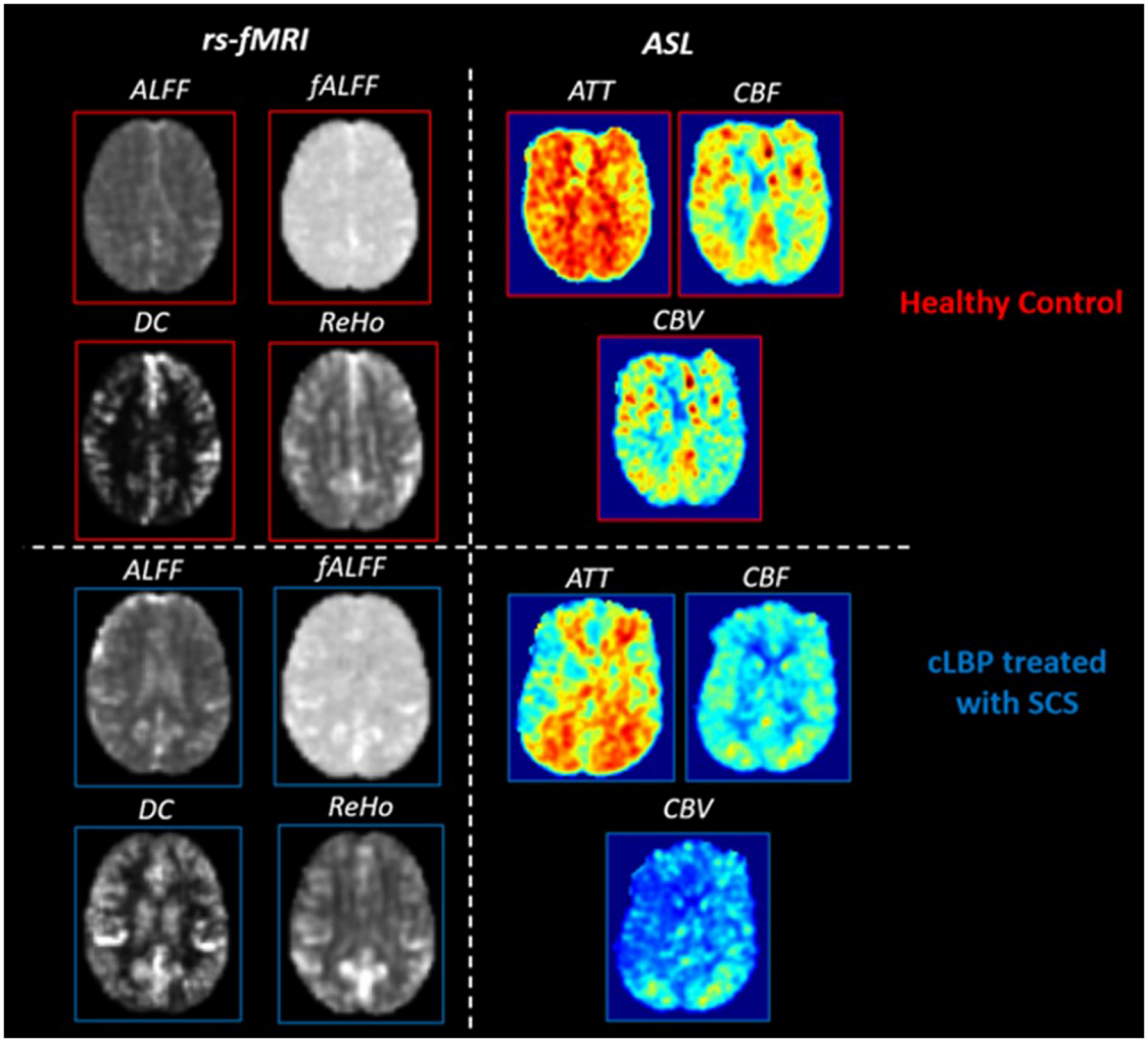
Representative rs-fMRI and PCASL parameter maps for healthy controls and cLBP patients implanted with SCS. Rs-fMRI measures: amplitude of low-frequency fluctuations (ALFF), fractional amplitude of low-frequency fluctuations (fALFF), degree of centrality (DC), regional homogeneity (ReHo). PCASL measures: arterial transit time (ATT), cerebral blood volume (CBV), and cerebral blood flow (CBF).

The representative quantitative maps for rs-fMRI and PCASL parameters for patients and HCs are shown in Figure 2. Table 4 summarizes the significant differences between cLBP patients implanted with SCS and HCs when assessing rs-fMRI quantitative measures (mean, SD, and 95% CI). Generally, significant differences showed an increase in rs-fMRI indices in patients as opposed to HCs, across several ROIs. The regions involved with initial pain sensation (S2) [fALFF: *t* = 3.75, *p* = 0.009], intensity coding (PS) [fALFF: *t* = 2.43, *p* = 0.039], and relay (VPL) [ALFF: *t* = 2.88, *p* = 0.018; fALFF: *t* = 4.15, *p* = 0.004] displayed increased neural activity. Additionally, the regions related to decision-making and stress/emotional response such as the dorsolateral prefrontal cortex (DLPC) [DC: *t* = 3.43, *p* = 0.007; ALFF: *t* = 2.84, *p* = 0.024], hippocampus (HIP) [ReHo: *t* = 2.44, *p* = 0.037; DC: *t* = 2.77, *p* = 0.024], and posterior cingulate cortex (PCC) [fALFF: *t* = 3.26, *p* = 0.01] showed increased neural activity. Finally, the hypothalamus (HT) [ALFF: *t* = 4.60, *p* = 0.001] also displayed increased neural activity, as indexed by rs-fMRI.

**Table 4:** Comparisons of pain ROIs using rs-fMRI measures in patients and healthy controls.

**ROI (rs-fMRI indices)**		**Mean (SD)**		**95% CI**		***T*-statistic**	***P*-value**
		**HC**	**SCS-cLBP**	**HC**	**SCS-cLBP**		
ReHo	HIP	0.84 (0.023)	0.93 (0.026)	(0.79, 0.9)	(0.87, 0.96)	2.44	0.037
DC	HIP	0.77 (0.052)	0.99 (0.058)	(0.65, 0.89)	(0.86, 1.12)	2.77	0.024
DC	DLPC	−0.25 (0.053)	0.01 (0.058)	(−0.37, −0.13)	(−0.12, 0.14)	3.43	0.007
fALFF	VPL	1.018 (0.008)	1.067 (0.008)	(1.001, 1.036)	(1.05, 1.09)	4.15	0.004
fALFF	PCC	1.07 (0.008)	1.11 (0.008)	(1.06, 1.09)	(1.09, 1.13)	3.26	0.01
fALFF	S2	0.92 (0.006)	0.96 (0.006)	(0.91, 0.94)	(0.94, 0.97)	3.75	0.009
fALFF	PS	1.033 (0.01)	1.074 (0.013)	(1.006, 1.06)	(1.04, 1.103)	2.43	0.039
ALFF	VPL	0.81 (0.02)	0.88 (0.02)	(0.76, 0.85)	(0.84, 0.92)	2.88	0.018
ALFF	HT	1.72 (0.1)	2.34 (0.11)	(1.49, 1.96)	(2.14, 2.65)	4.6	0.001
ALFF	DLPC	0.82 (0.026)	0.92 (0.29)	(0.76, 0.88)	(0.86, 0.99)	2.84	0.024

Results provided based on specific quantitative rs-fMRI maps and ROI, as mean (SD), and 95% CI.

Comparisons were completed with FDR correction, at p <0.05. ReHo, regional homogeneity; DC, degree of centrality; fALFF, fractional amplitude of low-frequency fluctuations; ALFF, amplitude of low-frequency fluctuations; HIP, hippocampus; DLPC, dorsolateral prefrontal cortex; VPL, ventral posterolateral thalamus; PCC, posterior cingulate cortex; S2, secondary somatosensory cortex; PS, posterior short gyrus; HT, hypothalamus.

#### Multi-delay PCASL

When assessing ATT, there was a significant increase in the supplementary motor area (SMA) (*t* = 2.47, *p* = 0.04), orbitofrontal cortex (OC) (*t* = 2.28, *p* = 0.05), and PS (*t* = 2.60, *p* = 0.03) (Table 5). There were no significant differences when comparing the CBF and CBV of patients and HCs for any ROIs.

**Table 5:** Comparisons of pain ROIs using PCASL measures in patients and healthy controls.

**ROI (PCASL indices)**		**Mean (SD)**		**95% CI**		***T*-statistic**	***P*-value**
		**HC**	**SCS-cLBP**	**HC**	**SCS-cLBP**		
ATT	SMA	1,064.50 (63.96)	1,156.94 (57.12)	(985.08, 1,143.92)	(1,086.02, 1,227.86)	2.47	0.04
ATT	OC	783.11 (96.5)	921.56 (100.4)	(663.29, 902.93)	(796.92, 1,046.25)	2.28	0.05
ATT	PS	908.89 (129.34)	1,164.47 (221.69)	(748.29, 1,069.49)	(889.2, 1,439.74)	2.6	0.03

Results provided based on specific quantitative PCASL maps and ROI, as mean (SD), and 95% CI. Comparisons were completed with FDR correction, at p <0.05. There were no significant differences in cerebral blood volume or flow between patients and healthy controls. Arterial transit time (ATT) was significantly increased in cLBP patients implanted with SCS in the supplementary motor area (SMA), orbitofrontal cortex (OC), and the posterior short gyrus (PS).

### Correlation of quantitative neuroimaging measures with clinical pain assessments

**Figure 3:**
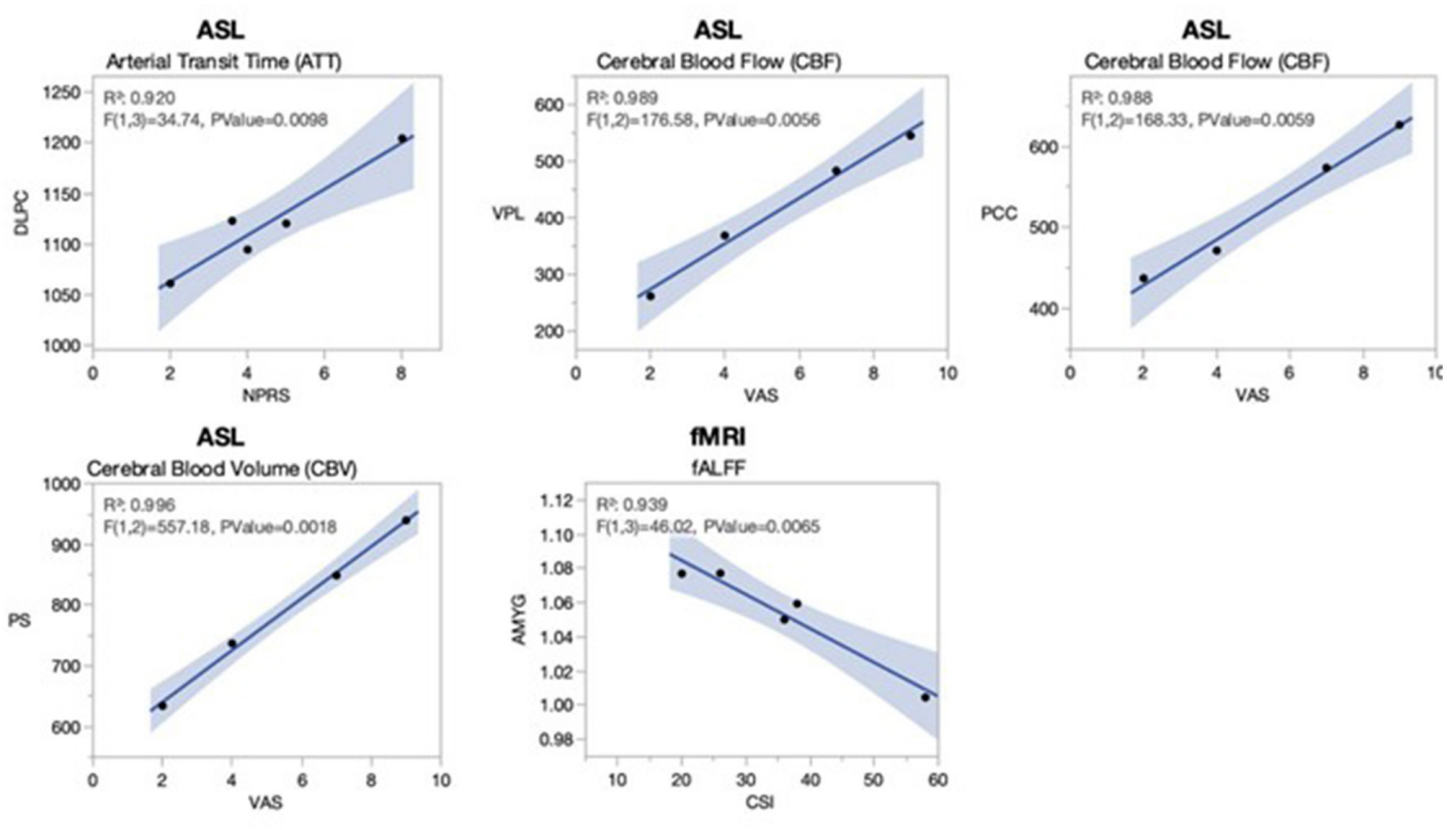
Significant linear associations between activation of pain regions and clinical measures in cLBP patients implanted with SCS. Clinical self-assessments of pain from the cLBP patients implanted with SCS were correlated using linear regression to the functional imaging maps for PCASL and rs-fMRI. Significant linear associations (all *R*^2^s > 0.92, all *p-*values <0.01) were observed for arterial transit time (ATT) of the dorsolateral prefrontal cortex (DLPC) with the numerical pain rating scale (NPRS); cerebral blood flow (CBF) in the ventral posterior lateral thalamus (VPL) with the visual analog scale (VAS); CBF in the posterior cingulate cortex with the VAS; cerebral blood volume (CBV) in the posterior short gyrus (PS) with the VAS; and fractional amplitude of low-frequency fluctuations (fALFF) in the amygdala (AMYG) with the central sensitization index (CSI). The pain VAS was only available for four of five patients. The gray shading represents a 95% confidence interval.

Figure 3 denotes significant correlations between rs-fMRI and PCASL quantitative measures and clinical scores of pain in the cLBP patients implanted with SCS. The fALFF in the amygdala (AMYG), a region involved in the fear and stress response, was negatively correlated with CSI scores (*R*^2^ = 0.939, *p* = 0.0065). The ATT of the DLPC, involved in the cognitive component of pain, was positively correlated with the NPRS scores (*R*^2^ = 0.92, *p* = 0.0098). The CBF showed significant correlations in two regions; VPL, the somatosensory relay of the thalamus, was positively correlated with VAS (*R*^2^ = 0.989, *p* = 0.0056); and PCC, associated with emotional appraisal, was also positively correlated with VAS (*R*^2^ = 0.988, *p* = 0.0059). Finally, the CBV in the PS, a nociceptive input region, was positively correlated with VAS (*R*^2^ = 0.996, *p* = 0.0018).

## Discussion

Certain regions of the brain are implicated in the experience of pain, and functional activity of these regions represents concerted processing of various dimensions, including pain intensity coding, sensation, information processing, cognitive and emotional appraisal, and response (Caylor et al., 2019; Davis and Moayedi, 2013). The present pilot study conducted a pain region-based analysis of rs-fMRI and PCASL in cLBP patients implanted with SCS and compared that to healthy control subjects. Clinical pain evaluations were highly correlated with the patient's rs-fMRI and PCASL region-based activity. We found significant differences between patients and HCs in regions involved with pain input, multimodal integration, and stress response. Additionally, there were several strongly significant correlations with evaluations related to pain rating and sensitization, despite the limited sample size of patients. This provides evidence of the capacity for PCASL and rs-fMRI-based imaging as quantitative measures for the beneficial effects of SCS on cLBP patients.

### Region-based comparison of patients to HCs

#### Primary pain sensation and nociceptive input

The fALFF and ATT of PS and the ALFF/fALFF of VPL thalamus were significantly higher in SCS patients than in HCs. The VPL thalamus plays a role in providing ascending sensory information from the body to S1, including the dorsal column medial lemniscus (light touch and proprioception) and spinothalamic tract (nociception and temperature) (Jones, 1998). Upregulation of spontaneous neural activity as indexed by the ALFF/fALFF in this region may be associated with increased nociceptive afferent input in our patients, as a result of central sensitization (Syr et al., 2014). The insular cortex has been shown to be activated in pain studies in both healthy controls and individuals with chronic pain (Labrakakis, 2023). More specifically, the posterior insula, which includes the PS, is involved in the processing of nociceptive input (Frot et al., 2014). Increases in ATT and fALFF in this region may represent changes in perfusion and spontaneous neural activity involved with pain coding when cLBP patients are implanted with SCS. Perfusion changes in brain regions are associated with the functional energy demand required for cellular respiration and more advanced processes such as long-term potentiation-like plasticity. PCASL measures such as ATT provide an index of this, which may be indicative of the functional changes associated with the adaptive plasticity associated with SCS compared to a cLBP patient who may not be receiving stimulation. Nevertheless, future investigations are warranted to study these functional/perfusion changes in the nociceptive inputs of cLBP patients implanted with SCS using pre-operative and post-operative imaging data.

#### Multimodal integration and modulation of pain

The above factors may similarly account for the differences observed in sensory association (S2) and frontal/limbic (DLPC, PCC, OC, and SMA) activity between our patients and HCs. S2 and SMA are both association cortices involved with the multimodal integration of inputs from motor, somatosensory, limbic, and insular regions (Wu et al., 2017; Cona and Semenza, 2017; Mehler et al., 2019). In particular, S2 has been implicated in the persistence of chronic pain states, whereas SMA may play a role in more cognitive aspects of pain modulation, such as attention shifts and motor imagery of movements in response to pain (Cona and Semenza, 2017). This suggests that increased SMA activity serves an adaptive role following the bottom-up effects of SCS on the cortex over time. DLPC and OC are other frontal cortex structures that have been greatly implicated in the cognitive and reward modulation of pain processing, respectively (Lorenz et al., 2003; Seminowicz and Moayedi, 2017; Becker et al., 2017). PCC has been associated with general emotional information processing, receiving input from the insula, limbic cortex, and DLPC (Vogt, 2014). Gross functional/perfusion changes in these structures in cLBP patients implanted with SCS may lead to improved multimodal integration and top-down adaptive processing of pain, as opposed to patients not receiving any spinal stimulation and HCs.

#### Stress response to chronic pain

Finally, ReHo and DC of HIP and ALFF of HT were increased in our cLBP patients implanted with SCS. The hippocampus and hypothalamic-pituitary-adrenal axis, along with other limbic and prefrontal regions, form a loop that regulates the stress response and subsequent cortisol release involved in chronic pain (Abdallah and Geha, 2017). This interconnection between chronic pain and stress is a likely cause as to why cLBP patients have upregulated functional activity in these regions, with acute pain leading to long-term stress, further exacerbating the disabling effects of chronic back pain (Hannibal and Bishop, 2015). Future studies are warranted to investigate the effects of SCS on the stress response in the brains of cLBP patients.

### Correlation of pain regions in cLBP patients implanted with SCS to clinical pain evaluations

The correlation of imaging-derived functional pain ROIs to clinical measures of pain in cLBP provides evidence of the utility of rs-fMRI and PCASL as measures of pain prognosis, along with quantifying the effects of SCS as a modulator of central pain processing. Despite a small patient population, analyses showed several strong statistically significant linear associations. VAS pain ratings were positively correlated with VPL CBF and PS CBV. As previously described, the VPL thalamus is associated with afferent nociceptive input and the insular PS with pain intensity coding (Afif et al., 2008; Ab Aziz and Ahmad, 2006). Both regions were also found to have increased rs-fMRI/PCASL measures when comparing cLBP patients to HCs. Therefore, we speculate that these regions, part of the input component of the pain Neuromatrix, are likely involved with unidimensional pain perception and rating. Patients with a higher pain rating had increased activity in these regions, suggesting differences in the responsiveness to SCS and the ability of imaging to prognosticate individual responses to SCS.

As ATT in the DLPC increased, there was a positive correlation in the NPRS. Similarly, there was a positive correlation between pain VAS and CBF in the PCC. Elevated cerebral blood flow to the PCC has been associated with self-referential pain catastrophizing, particularly as a component of the default mode network (DMN) (Lee et al., 2018). The DMN, which includes the PCC, DLPC, and other regions, is associated not only with passive brain processing but also has various cognitive and emotional appraisals of chronic pain. Therefore, it is possible that increased functional activation as indexed by perfusion in the PCC and DLPC in our patients may also lead to increased unidimensional rating of pain due to modulation of the DMN in cLBP patients implanted with SCS.

Finally, there was a statistically significant negative correlation between fALFF of the amygdala and CSI. The amygdala has an important function in the emotional and fear aspects of pain processing in cLBP, with it being associated with individual differences in emotional pain facilitation (Gandhi et al., 2020; Mao et al., 2022). A recent study found that reduced resting-state functional connectivity in the amygdala was correlated with mechanical pain rating in low-back-related leg pain patients (Wang et al., 2023). In our patients with SCS we observed the opposite effect, which suggests that the bottom-up effects of SCS contribute to the improvement of the emotional response in cLBP. Maladaptive anticipation and fear of pain in cLBP likely contribute to central sensitization in patients, with individual differences and the effects of SCS on the amygdala's functional state being a potential quantitative measure of the reversal of this process.

Rather than relying on subjective and qualitative measures of pain perception via the aforementioned pain scales, PCASL and rs-fMRI have the potential to be utilized as quantitative measures to evaluate treatment effectiveness. However, since we did not obtain baseline imaging of our cLBP patients before SCS, we cannot conclude whether changes in processing may be due to SCS, chronic pain experienced by patients, or both. Future studies are warranted to assess pre- vs. post-SCS changes in functional activation in cerebral pain regions, along with how these correlations change over this period.

### Limitations and future directions

The present study has limitations. First, we have a lack of baseline preoperative imaging of cLBP patients. This would serve as a comparator to the effects of SCS stimulation in this patient population, rather than relying strictly on comparisons to healthy controls. However, as a feasibility study, our purpose was to illustrate that rs-fMRI and PCASL from 3T MRI are effective modalities for distinguishing between healthy subjects and patients implanted with SCS. Additionally, these modalities are associated with clinical scores of pain in cLBP patients who have been implanted with SCS. Future studies are warranted to assess changes in functional imaging pre- vs. post-SCS in cLBP patients. This may even be used as a screening tool to eventually predict response to SCS by correlating the change in imaging measures with clinical response. By the same token, a comparison of MRI measures using the SCS “on” vs. “off” settings would provide an assessment of the immediate, “online” effects of SCS in cLBP patients, and future investigations are warranted for this understudied effect. Second, our limited sample size may restrict the reproducibility of results, and we did not control for age or sex. However, we utilized FDR correction, and correlation analyses were highly significant. Additionally, our patients may have other comorbidities, medications, and individual differences in their SCS parameters and timeline of obtaining SCS relative to cLBP onset. Nevertheless, functional imaging of pain regions in cLBP patients implanted with SCS is a greatly underexplored area of research that warrants further investigation, assessing differences in pain states based on SCS parameters and paradigms (e.g., paresthesia-based vs. burst vs. 10 KHz) and timeline of changes based on duration of SCS, using a larger sample size. Finally, although our patients had differing responses to SCS in regard to pain control, our correlation analyses do not provide a direct comparison of patients who obtain excellent pain control vs. those who do not. Future studies are warranted to compare these groups using a larger, predetermined group of patients, as this would shed light on the central pain mechanism as well as potentially improve the presurgical indications of SCS based on the neural activity of cLBP patients.

### Conclusion

This pilot study explored pain states in cLBP patients implanted with SCS using rs-fMRI and PCASL, safely derived from 3T MRI. Changes in rs-fMRI and PCASL measures were observed when comparing patients to healthy control subjects, and activation of several pain regions was associated with clinical scores of pain rating and sensitization. Specifically, the frontal and limbic cortex structures, VPL thalamus, and posterior short gyrus of the insula were all associated with changes in pain rating and/or sensitization. The utilization of advanced imaging techniques in cLBP patients implanted with SCS may be eventually developed as a quantitative tool in patients and is vital for monitoring other conditions, presurgical planning, and the study of chronic pain and its neuromodulation.

## Data Availability

The datasets presented in this article are not readily available because anonymized data is available upon direct request. Requests to access the datasets should be directed to mahdi.alizadeh2@jefferson.edu.
